# Ultrasonic Phased Array Compressive Imaging in Time and Frequency Domain: Simulation, Experimental Verification and Real Application

**DOI:** 10.3390/s18051460

**Published:** 2018-05-08

**Authors:** Zhiliang Bai, Shili Chen, Lecheng Jia, Zhoumo Zeng

**Affiliations:** State Key Laboratory of Precision Measurement Technology and Instrument, Tianjin University, Tianjin 300072, China; zhl_bai@tju.edu.cn (Z.B.); jialecheng@tju.edu.cn (L.J.); zhmzeng@tju.edu.cn (Z.Z.)

**Keywords:** ultrasonic phased array, compressive sensing, image reconstruction, time and frequency domain, engine cylinder cavity

## Abstract

Embracing the fact that one can recover certain signals and images from far fewer measurements than traditional methods use, compressive sensing (CS) provides solutions to huge amounts of data collection in phased array-based material characterization. This article describes how a CS framework can be utilized to effectively compress ultrasonic phased array images in time and frequency domains. By projecting the image onto its Discrete Cosine transform domain, a novel scheme was implemented to verify the potentiality of CS for data reduction, as well as to explore its reconstruction accuracy. The results from CIVA simulations indicate that both time and frequency domain CS can accurately reconstruct array images using samples less than the minimum requirements of the Nyquist theorem. For experimental verification of three types of artificial flaws, although a considerable data reduction can be achieved with defects clearly preserved, it is currently impossible to break Nyquist limitation in the time domain. Fortunately, qualified recovery in the frequency domain makes it happen, meaning a real breakthrough for phased array image reconstruction. As a case study, the proposed CS procedure is applied to the inspection of an engine cylinder cavity containing different pit defects and the results show that orthogonal matching pursuit (OMP)-based CS guarantees the performance for real application.

## 1. Introduction

Ultrasonic phased array is one of the most widely used imaging modalities in current industrial non-destructive evaluation (NDE) due to its increased flexibility, faster detection speed, higher inspection quality and radiation-free operation [1]. With programmable time delays, a single linear ultrasonic array can be used to undertake various inspections and produce real-time 2D images, which brings efficiency in the structural health monitoring (SHM) of some industrial components with complex geometries [2,3].

Performed digitally, it is required that the analog signals first be sampled in the inspection, which is confined to traditional Nyquist-Shannon sampling limitation. Although the Nyquist rate is defined as twice the highest frequency component in the signal, in practice, oversampling (generally 4 to 10 times that of the transducer central frequency) is implemented in order to improve resolution, reduce noise and avoid aliasing. Furthermore, as image techniques developing and the requirement of inspection accuracy improving, the number of elements involved in phased array typically rises. Consequently, the A-scan lines in an image increase, leading to a huge amount of data collection from the system front-end, eventually exerting pressures on data acquisition sensors. Therefore, it is imperative to develop compression methods to effectively reduce the sampling rate.

In recent years, Compressive Sensing (CS) has become a challenging field that has driven a lot of research interests in SHM applications, such as, the sparse recovery optimization in wireless sensor network [4], the mode separation in Lamb wave-based long-range damage detection [5], the reliable estimation of vehicular position in general traffic scenarios [6] and the recovery of the lost data in civil SHM [7]. Exploiting a priori knowledge that many natural measurement signals admit a sparse representation on proper basis or a redundant dictionary, CS provides one approach to achieve qualified signal reconstruction with fewer measurements compared with Nyquist sampling criteria [8]. Contrary to the traditional compressions, the CS encoder is based on a very simple and extremely low-power hardware, while most of the complexity and energy requirements are transferred to the decoding stage [9]. 

Up till now, CS has found increasing interests in the ultrasonic inspection community, which generally falls into two categories: medical ultrasound of diagnostic sonography [10,11,12,13,14] and guided wave-based NDE applications [15,16,17,18,19,20]. For the former, by treating ultrasound signals within the FRI framework, Wagner et al. generalized the concept of compressed beamforming following the spirit of Xampling [10]. Lorintiu et al. presented a CS-based reconstruction of 3D ultrasound data using dictionary learning and line-wise subsampling, which confirmed a better performance than conventional fixed transforms [11]. Foroozan et al. employed wave atom dictionary as a low dimension projection and the robust Capon beamformer was combined with CS, instead of using the delay-and-sum method [13]. In the case of ultrasonic NDE, López et al. analyzed the application of CS techniques in order to achieve faster scanning in the ultrasonic imaging of cargo containers [15]. Di et al. used a random sampling scheme based on CS to minimize the number of points at which the field is measured [16]. Mesnil et al. proposed a reconstruction technique to estimate the location of sources and structural features interacting with the waves from a set of sparse measurements [17]. Wang et al. applied a dictionary algorithm on sparse representation for Lamb-wave-based damage detection [18]. In all cases, some only apply CS in ultrasound single signals [11,12,17,18] while others extend it to ultrasound images.

On the other hand, there are several reports concentrating on the use of CS in frequency domain recently. Chernyakova et al. [21] demonstrated on in vivo cardiac data that reductions up to 1/28 of the standard rates are possible, using only a portion of the beamformed signal’s bandwidth. Their study was also extended to the 3D beamforming in frequency domain and satisfactory results can be obtained [22]. Mishra et al. [23] demonstrated a significant superiority of frequency domain CS reconstruction but no further explanation was offered. 

**Figure 1:**
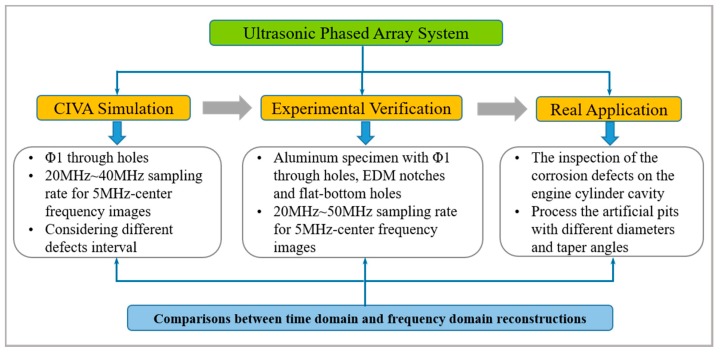
The simplified architecture of the proposed compressive sensing (CS)-based scheme.

Compared with a large number of literatures on medical ultrasound or guided wave-based NDE fields, the CS applications on ultrasonic phased array are still inadequate and deserve more attentions. In this work, we applied the CS framework to a general phased array image reconstruction in both time and frequency domain, aiming at verifying the feasibility and potentiality of CS in this field. Employing the peak signal to noise ratio (PSNR, in dB) and the structural similarity (SSIM) as the evaluation criteria, images of different sampling rate (SR) are recovered under various compression ratios (CR). The results comparisons between time and frequency domain are presented and the causes behind the performances are explained, especially the superiority in frequency domain. In order to investigate the influence of SR level on the recovery performance, we compare the PSNR of different SRs when keeping the measurement points the same. Besides, considering the presence of close defects in real-world components, the recoveries when the distance of defects is different are also included, accompanied by results analysis. Here, the Orthogonal Matching Pursuit (OMP) was utilized to reconstruct all images. Simulations based on CIVA platform, experiments on three kinds of artificial flaws (through-holes, electrical discharge machining (EDM) notches and flat-bottom holes) and real application of engine cylinder cavity inspection are exploited to demonstrate the performance of the proposed framework. Figure 1 illustrates the simplified procedure of the proposed CS-based scheme for ultrasonic phased array image reconstruction in time and frequency domain.

The paper is structured as the following: Section 2 presents an introduction of the CS theory, including sparsity of transform coding, measurement with incoherence and reconstruction via optimization. Section 3 shows the simulated study based on images obtained from CIVA. Section 4 contains the details of experiment setup and of the implementation process. Finally, Section 5 summarizes the main findings of this work and ends the paper.

## 2. Overview of CS

CS, as a novel sampling paradigm that goes against the common wisdom in data acquisition, successfully combines sampling and compression together. To make this possible, two fundamental prerequisites play a critical role: sparsity, which relates to the signal essence; and incoherence, which associates with sensing modality.

### 2.1. Sparsity of Transform Coding

Although many natural signals are not sparse in their own domain, they admit a sparse representation when expressed in a proper basis. Mathematically speaking, given a basis or dictionary $\Psi \in {\mathbb{R}}^{N\times N}$, we represent vector $f\in {\mathbb{R}}^{N}$ as a linear combination of a few atoms:

(1) $$f=\Psi x=\sum_{i=1}^{N}x_{i}\psi_{i}$$

where $x_{i}$ are coefficients and $x_{i}=\langle f,\psi_{i}\rangle =\psi_{i}{}^{T}f$. If at most *K* components of $x_{i}$ are nonzero, we say that f is *K*-sparse. In practice, there is a strong possibility that these coefficients are close to 0, rather than equal to 0, we regard it as compressible. At present, the commonly used sparse transformation bases include Discrete Fourier transform (DFT), Wavelet basis, redundant dictionaries [24] and adaptive sparse decomposition.

In the following simulations and experiments, DFT, the most typical orthogonal transformation is employed. It can be expressed as

(2) $$f=\sum_{n=0}^{N-1}x(n)e^{-j2\pi kn/N},\;0\leq k\leq N-1$$

### 2.2. Measurement with Incoherence

With consideration of noise, this section begins with a measurement system that acquire *M* linear measurements data:

(3) y=Φf+n

where ***n*** is an unknown error term and $\Phi \in {\mathbb{R}}^{M\times N}$ can play a role of dimensionality reduction, for *M* is typically much smaller than *N*.

For a sparse or compressible signal f, we can rewrite Equation (3) as

(4) y=Ax+n

where A=ΦΨ is denoted as the sensing matrix. To guarantee the robustness and validity of the *K*-sparse signal recovery, a sufficient condition is that matrix **A** satisfies the restricted isometry property (RIP) of order *k* if there exists a $\delta_{k}\in (0,1)$ such that [8]

(5) $$(1-\delta_{k}){\left\|x\right\|}_{2}^{2}\leq {\left\|Ax\right\|}_{2}^{2}\leq (1+\delta_{k}){\left\|x\right\|}_{2}^{2}$$

holds for all *K*-sparse vectors x.

It has been proved that an equivalent condition of RIP is the incoherence between Φ and Ψ [25]. The coherence is defined as

(6) $$\mu (\Phi ,\Psi )=\sqrt{N}\cdot \underset{1\leq k,j\leq N}{\max}\left|\langle \varphi_{k},\psi_{j}\rangle \right|$$

where φ and ψ represent any two elements of Φ and Ψ. The less correlated elements they have, the smaller coherence will be get. It is shown that random matrices with Gaussian or Bernoulli distributions can satisfy the RIP with high probability [26]. 

### 2.3. Reconstruction via Optimization

If the original signal f is rationally sparse and the RIP holds, it is possible to accurately recover ***x*** by convex programming:

(7) $$\hat{x}=\arg\min{\left\|x\right\|}_{1}\;\mathrm{subject}\;\mathrm{to}\;{\left\|Ax-y\right\|}_{2}\leq \varepsilon$$

where ${\left\|x\right\|}_{1}=\sum \left|x_{i}\right|$ is the $l_{1}$ norm, ${\left\|\cdot \right\|}_{2}$ represents the standard Euclidean norm and $\varepsilon^{2}$ is a likely upper bound on the noise power ${\left\|n\right\|}_{2}^{2}$.

For the sake of computing efficiency, one of the most frequently used greedy algorithms, that is, OMP, will be adopted in our work. The mathematical description of OMP is formally defined in Algorithm 1 [27].
**Algorithm 1** Orthogonal Matching Pursuit (OMP)**Input:** A signal $y\in {\mathbb{R}}^{N}$, a matrix $A\in {\mathbb{R}}^{M\times N}$. **Initialize:** Set the support set $\Omega_{0}=\emptyset$, the residual error $r_{0}=y$ and put the counter k=1. **Identify:** Find a column $a_{n}$ from **A** that most correlates with the residual error and record the correlation coefficient:$$n_{k}\in \arg\underset{n=1,2,\ldots N}{\max}\left|\langle r_{k-1},a_{n}\rangle \right|,\;\Omega_{k}=\Omega_{k-1}\cup \left\{n_{k}\right\}$$**Estimate:** compute the best approximating coefficients:$$x_{k}=\arg\min_{x}{\left\|y-A_{\Omega_{k}}x\right\|}_{2}$$**Iterate:** Update the residual and counter: $$r_{k}=y-A_{\Omega_{k}}x_{k},\;k=k+1$$**Until:** Stopping criterion holds. **Output:** The vector $\hat{x}$ with components $\hat{x}(n)=x_{k}(n)(n\in \Omega_{k})$ and $\hat{x}(n)=0$ otherwise.

## 3. Simulation Results and Discussion

To justify the feasibility of CS framework and to see how it helps for data compression in ultrasonic phased array imaging, a simulation procedure based on CIVA software (Version 9.0, Paris, France) is provided in this section.

### 3.1. Simulation Settings

Developed by CEA (the French Atomic Energy Commission) since the early 90’s, the CIVA gathers most influential parameters and advanced modeling tools into an expertise platform, making it more and more widely used in the industrial NDE fields (Ultrasound, Eddy Current, Radiography, ...) [28,29]. 

**Figure 2:**
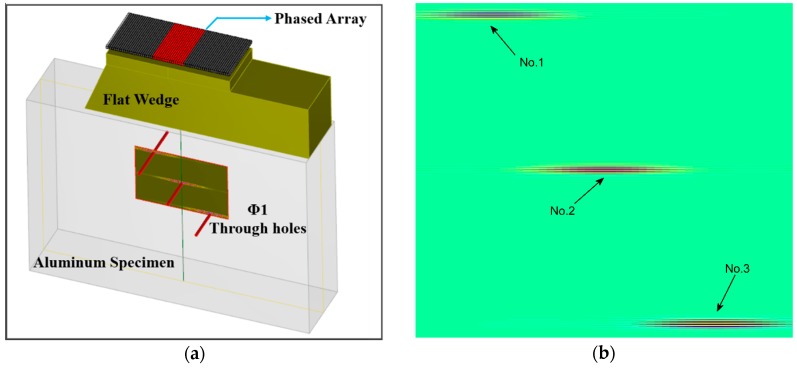
(**a**) The simulation setup using CIVA software; (**b**) the original image of 10 mm-space through holes.

As Figure 2a shows, we simulated an array aperture comprising a 64-element linear phased array transducer of 5 MHz center frequency. A 20 mm thickness flat wedge was used to eliminate the potential near-field influence. More detailed parameters are shown in Table 1. The aluminum specimen with three 1 mm diameter through holes was inspected using plane B-scan modality. The horizontal and vertical space were set as 10 mm, 8 mm, 6 mm, 4 mm and 2 mm, respectively. In the following implementations, 16 element chips are excited each time, resulting 49 sequences contained in each image. Figure 2b is the original image of 10 mm-space through holes.

### 3.2. Time Domain Reconstructions

In the simulation, we employed the discrete Fourier basis as sparse basis and the Gaussian random matrix as sensing matrix, exploiting their properties of calculation efficiency and implementation simplicity. Due to the restriction of hardware implementation, in our work, various CR levels were obtained by removing some parts of the original samples, as many reports generally used [12,30]. Here, the CR is determined by the ratio:

(8) $$\mathrm{CR}=(1-\frac{M}{N})\times 100\%$$

where *M* is the measurement points in the given domain and *N* represents the signal length of each sequence.

Quantitative evaluation of the proposed CS method was performed with respect to the recovered two-dimensional image. To quantify the reconstruction performance, we mainly used the PSNR, given by:

(9) $$\mathrm{PSNR}=10\mathrm{lg}(\frac{255^{2}}{\mathrm{MSE}})$$

Given a m×n original image *I* and its reconstructed version *R*, the mean squared error (MSE) measures the average of the squares of the deviations between *I* and *R*:

(10) $$\mathrm{MSE}=\frac{1}{mn}\sum_{i=0}^{m-1}\sum_{j=0}^{n-1}{\left[I(i,j)-R(i,j)\right]}^{2}$$

Although a higher PSNR generally indicates a better performance of CS algorithms, PSNR is just a kind of approximation to human perception of recovery quality. In our work, comparison between the original and reconstructed images is also performed by calculating the perception-based structural similarity (SSIM) index [31]:

(11) $$\mathrm{SSIM}(P,Q)={\left[l(P,Q)\right]}^{\alpha }\cdot {\left[c(P,Q)\right]}^{\beta }\cdot {\left[s(P,Q)\right]}^{\gamma }$$

where l(P,Q), c(P,Q) and s(P,Q) represent luminance comparison function, contrast comparison function and structure comparison function between images *P* and *Q*, respectively. α>0, β>0 and γ>0 are parameters used to adjust the relative importance of the three components. The resultant SSIM index is a decimal value between 0 and 1, and value 1 is only reachable in the case of two identical sets of data.

In any given detection area, different sampling rate (SR) means different signal length of the time traces. Therefore, the image compression potentiality was explored by varying the SR levels and 20 MHz, 25 MHz, 30 MHz, 40 MHz SR were considered in the simulation. The 40 MHz SR results in an overall number of 1168 real-valued samples (N = 1168), the data lengths of other SRs are proportional. To eliminate the negligible difference caused by the randomness of Gaussian matrix, each PSNR or SSIM data point averaged out the results of 100 runs. 

**Figure 3:**
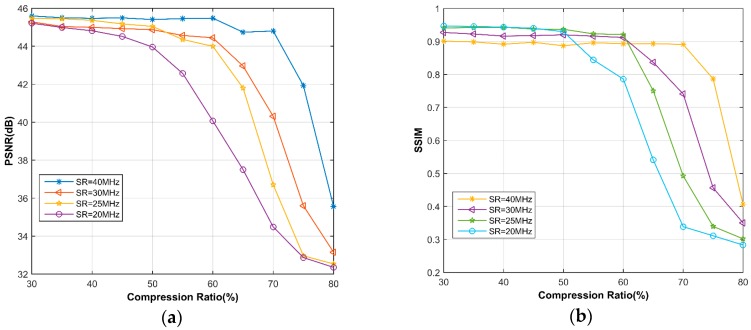
Comparative (**a**) Peak signal to noise ratio (PSNR), (**b**) Structural similarity (SSIM) between different sampling rates (SRs) in the time domain, using simulated data.

Figure 3 shows the comparative results as a function of CR. It is clear that the reconstruction quality generally decreases as CR rises. In the case of 65% CR or above, the reduction of SR leads to lower PSNR as well as SSIM. When SR 25 MHz, the impact of different SRs on the recovery performance is relatively small, with the PSNR > 43.99 dB and the SSIM > 0.8931 at 60% CR.

**Figure 4:**
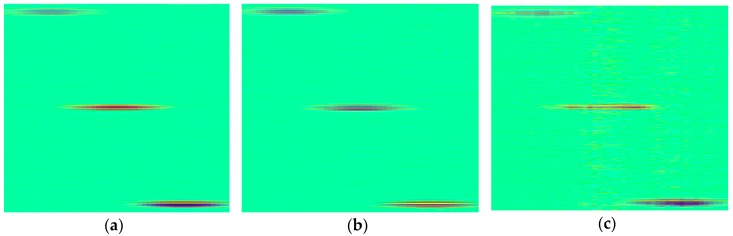
The recovery results of 25 MHz SR simulated image in time domain, (**a**) Compression ratio (CR) = 50%, PSNR = 45.05 dB; (**b**) CR = 60%, PSNR = 43.99 dB; (**c**) CR = 70%, PSNR = 36.71 dB.

Reconstructed images obtained by the proposed CS method, using 50%, 40%, 30% samples per image line in time domain, are shown in Figure 4. Here, the SR is 25 MHz. As can be seen from comparison with the original image, shown in Figure 2b, the recovery results when CR is 50% or 60% are in good agreement with our expectation: three defects are clearly preserved and almost no reconstruction errors exist. However, satisfactory performance cannot be realized if CR is 70%, although the defect echoes are roughly recognizable.

Notably, in the case of 25 MHz SR and 60% CR, the measurement points used for gratifying reconstruction are as many as that of 10 MHz SR original sample (25×(1−60%)=10). As the 5 MHz-center frequency pulses are employed and its −6 dB bandwidth is 50%, the minimum sampling rate is 15 MHz according to the Nyquist theory. The fact indicates that simulated images can be perfectly recovered in time domain from fewer samples than the Nyquist sampling limitation. Likewise, for 20 MHz SR and 50% CR, the conclusion is the same, with PSNR being 43.96 dB and SSIM being 0.9296.

As can be readily seen, either the lower SR or higher CR causes the reconstructed images to distort, because both cases mean the less measurement points (M) used for recovery. Next, we concentrate our interest on finding out whether the SR level has impact on the reconstruction performance. In Table 2 we compare the PSNR of different SRs when keeping the measurement points the same. Contrary to earlier results, for the measurement points up to 260, PSNR decreases with the increase of SR. This observation illustrates that the reconstruction accuracy of OMP is likely affected by the ratio of M/N, especially when M is comparatively small. This can be explained by the fact that for low M/N, the structure of sensing matrix becomes ill-balanced, which has an adverse impact on the recovery. After increased to 280, we can see that there is no obvious PSNR distinctions between different SRs.

**Figure 5:**
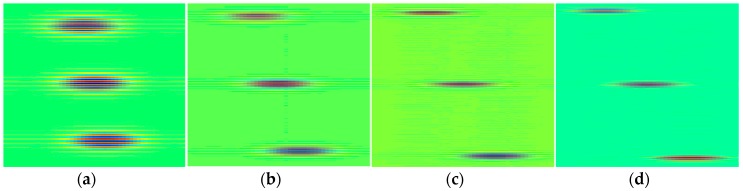
The recovery results of 25 MHz SR (CR = 60%) images in time domain when the distance of defects is different (**a**) D = 2 mm, PSNR = 45.62 dB; (**b**) D = 4 mm, PSNR = 45.33 dB; (**c**) D = 6 mm, PSNR = 45.17 dB; (**d**) D = 8 mm, PSNR = 44.25 dB.

In ultrasonic phased array NDE, there are always defects that are very close to each other. Therefore, it is indispensable for CS to distinguish these adjacent flaws in the reconstructed image. Using 25 MHz SR and 60% CR, Figure 5 presents the recovery results when the distance of defects (D) is different. As can be seen, the reconstructions are all quite qualified, which shows that the proposed OMP-based CS guarantees the reconstruction accuracy in terms of close defects. Actually, smaller defects distance brings about better PSNR.

### 3.3. Frequency Domain Reconstructions

**Figure 6:**
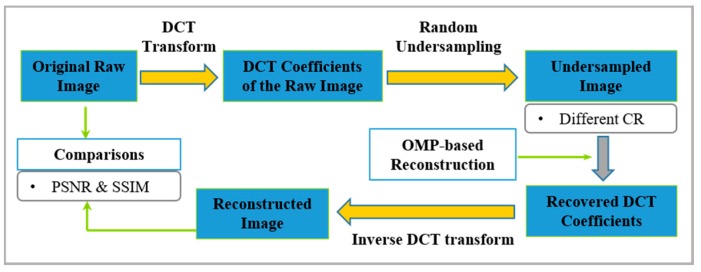
The sketch of the procedure used to reconstruct phased array images in frequency domain.

As a next step, we implemented reconstructions in frequency domain on simulated array images. Here, the Discrete Cosine transform (DCT), rather than the DFT, is employed in our work. The DCT only retains the cosine components of DFT, so the operation is more efficient and simplified. In this work, we use DCT coefficients to conduct CS recovery and the image in time is obtained by performing an inverse DCT transform. Figure 6 provides a sketch of the procedure used to reconstruct the undersampled phased array images in frequency domain. The sparse basis and sensing matrix exploited in the reconstructions are same as the previous section.

**Figure 7:**
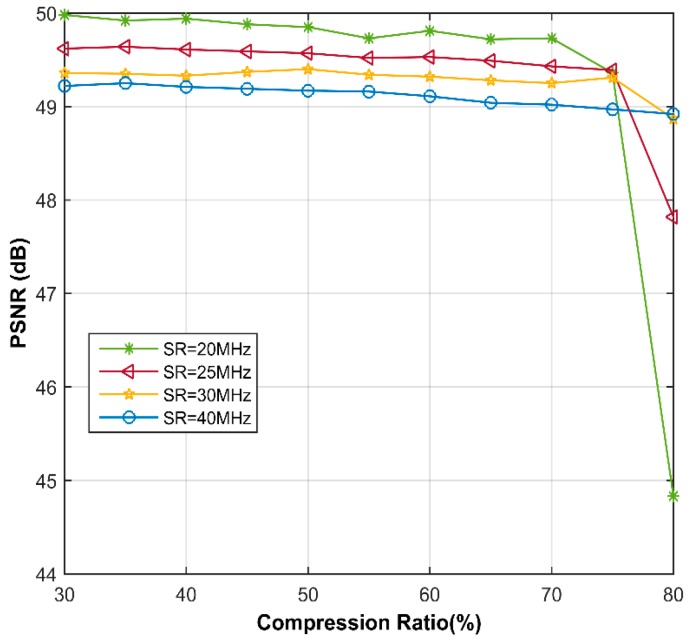
Comparative PSNR between different SRs in frequency domain, using simulated data.

**Figure 8:**
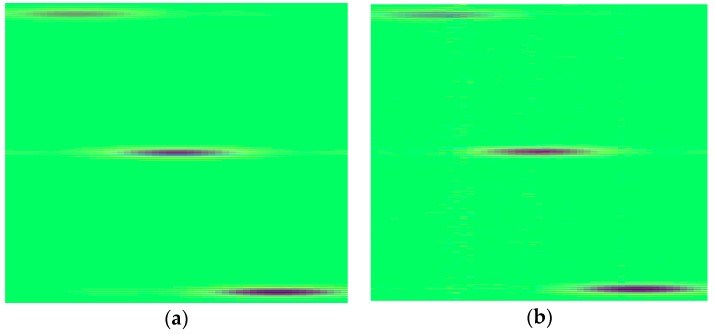
The recovery results of 20 MHz SR simulated image in frequency domain, (**a**) CR = 70%, PSNR = 49.73 dB; (**b**) CR = 80%, PSNR = 44.83 dB.

In Figure 7, the PSNR values are drawn as a function of CR and for different SRs. As an initial observation, we can note that the recovery performance in frequency domain is highly better than that in time domain. Except for 20 MHz SR, the PSNR values at 80% CR are all higher than 47 dB, which is better than any result in time domain. In addition, the SSIMs of all the reconstructed images in presence of frequency domain are very close to 1, so we would not present here. Contrary to the situation in time domain, lower SR lends to better PSNR when CR 70%, even if the differences are less than 1 dB. From the recovery images shown in Figure 8, it can be say that almost no error occurs in the reconstruction, using only 20% samples.

**Figure 9:**
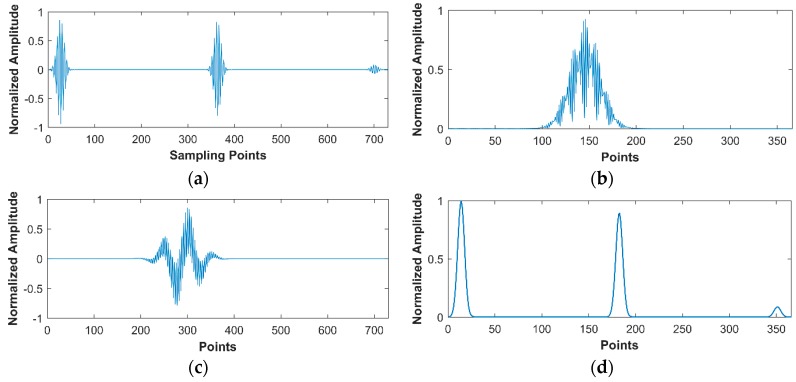
(**a**) Extracted A-scan signal from 25 MHz SR image; (**b**) the Discrete Fourier Transform (DFT) spectrum of (**a**); (**c**) the Discrete Cosine Transform (DCT) coefficients of (**a**); (**d**) the DFT spectrum of (**c**).

To explain the superiority of the frequency domain CS, we investigate the sparsity of the one-dimensional time trace as well as its DCT coefficients, through expressing them in the discrete Fourier basis. An arbitrary A-scan, shown in Figure 9a, is extracted from 25 MHz SR image and Figure 9b shows its corresponding Fourier spectrum. In frequency domain CS, the DCT coefficient of A-scan signal plays the role of input signal and is shown in Figure 9c,d is the corresponding DFT spectrum. We manually set a threshold to pick out the main elements, that is, only the values that are bigger than 1% of the maximum can be designated as “Information Value (IV)”. The IV of raw A-scan signal is 87, and for DCT coefficients, it is 60. The calculated results indicate that the DCT coefficient is sparser than the original time domain signal, that is, the excellent performance in frequency domain benefits from its higher sparsity.

**Figure 10:**
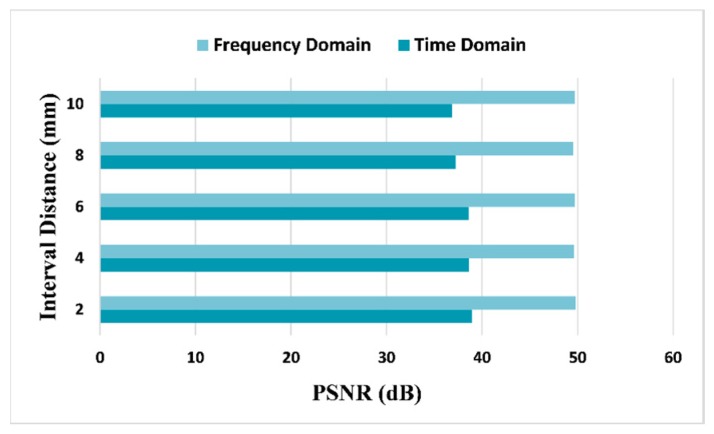
Comparative PSNRs between different defects interval spaces, using 25 MHz SR in time domain and 20 MHz SR in frequency domain, and CR is 70% for both domains.

Figure 10 considers different situations vary in defects interval distance and compares the PSNRs in two domains when keeping 70% CR. We note that the results in frequency domain are at least 10.8 dB higher than that in time domain, which confirms the capability of frequency domain CS in various defects cases.

To conclude, using samples less than the minimum requirements of the Nyquist theorem, both time domain and frequency domain CS verify their abilities to accurately reconstruct ultrasonic phased array simulated images. In addition, CS in frequency domain allows one to obtain a more satisfactory performance due to its inherent higher sparsity.

## 4. Experimental Results and Discussion

### 4.1. Apparatus, Real-Time Images and Algorithm Parameters

For experimental testing, the lab setup includes a standard PC, a commercial ultrasonic phased array detector (Multi 2000, Paris, France), a 5 MHz linear array with 64 elements and specimen with artificial defects. We particularly chose devices that share the same parameters with simulation for ease of performance comparison. The phased array was placed on a 20 mm-depth Plexiglas wedge to protect the probe from wearing excessively. Linear scanning was exploited in the inspection and all the signals were sampled at 20 MHz, 25 MHz, 33 MHz and 50 MHz, respectively.

**Figure 11:**
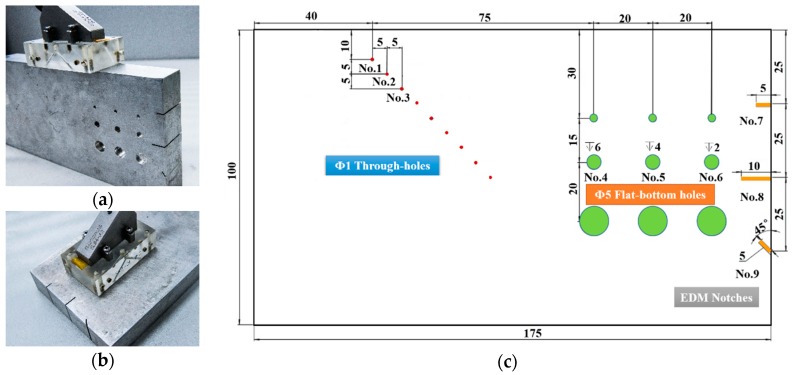
(**a**) The inspection of through-holes and electrical discharge machining (EDM) notches; (**b**) The inspection of flat-bottom holes; (**c**) The specimen geometry and defects distribution.

The test specimen was manufactured from Aluminum with three kinds of defects. Measurements of through-holes and electrical discharge machining (EDM) notches were performed as shown in Figure 11a. Figure 11b shows how flat-bottom holes were inspected. The specimen geometry and details of defects distribution are indicated in Figure 11c. 

**Figure 12:**
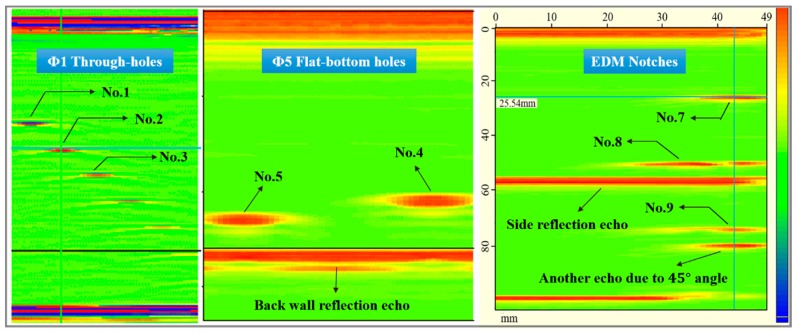
The real-time measurements of three kinds of defects.

Part of real-time images of 50 MHz SR are shown in Figure 12. For through-holes, it is clear that the signal intensity decreases with the transmission distance. For flat-bottom holes and EDM notches, although a strong back wall echo or side reflection echo is quite obvious, the pre-machined flaws are all well detected. And, more remarkably, the measured depths coincide very well with the actual positions. All kinds of defects recorded by the linear array ensure an overall understanding of the detection, location and quantitatively sizing of defects. 

### 4.2. Time Domain Reconstructions

**Figure 13:**
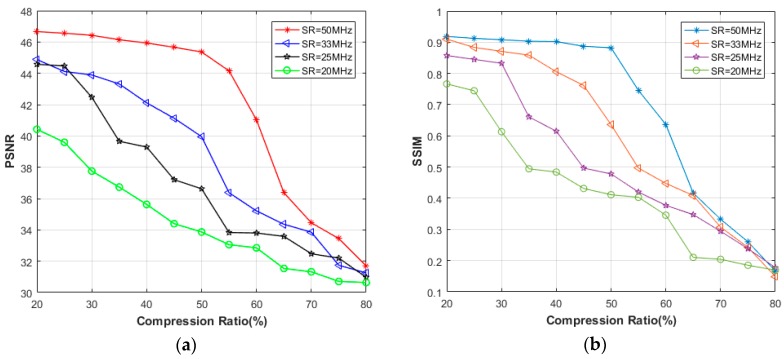
Comparative (**a**) PSNR, (**b**) SSIM between different SRs in time domain, using experimental through-holes data.

Similar as the simulation, the images sampled from 20 MHz, 25 MHz, 33 MHz and 50 MHz SR were considered in the experiment. In our work, 16 elements were activated each time, leading to 49 sequences per image (64 − 16 + 1 = 49). We first take the through-holes as an example to perform time domain recovery. For the ease of calculation and comparison, 512 points containing the main part of defects zone was intercepted in 50 MHz SR. The average results of 100 runs are illustrated in Figure 13. Considering the complexity of real experiment, we first note that the overall performances are worse than with the simulated images. To be exact, when CR is 60%, the PSNRs at least reduce 5 dB except for 50 MHz SR. For SSIMs, the negative effects are more exposed. In addition, the performances are more liable to affected by the SR level. For 50 MHz SR, the results are relatively satisfactory when CR is not more than 60%. By contrast, when the SR drops to 20 MHz, the reconstruction is just barely good in 20% CR.

**Figure 14:**
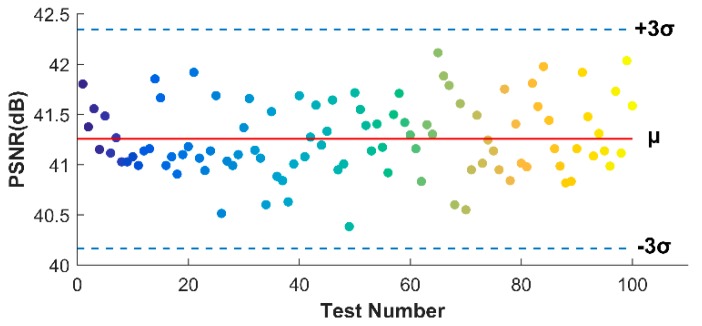
Distribution of 100 OMP runs based on 3*σ* criterion of reliability theory in time domain, using 50 MHz SR experimental through-holes data when CR is 60%.

To clearly show the statistic results, the scattered point distribution of 100 releases testing (50 MHz SR, 60% CR) is shown in Figure 14, combined with the 3*σ* (*σ* is the standard deviation) criterion of reliability theory. It is clear that all the PSNR results lie within three standard deviations on either side of *μ* (the mean value), which confirms the reliability of OMP algorithm. By calculating all the standard deviation values of different SRs and CRs, we show in Table 3 that, except for some failures when SR is low or CR is high, most of the situations can successfully fit with the 3*σ* criterion.

**Figure 15:**
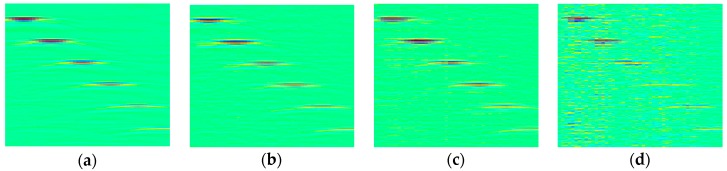
(**a**) The original experimental through-holes image of 50 MHz SR and the reconstructed results in time domain; (**b**) CR = 50%, PSNR = 45.36 dB; (**c**) CR = 60%, PSNR = 41.25 dB; (**d**) CR = 70%, PSNR = 34.45 dB.

Figure 15a is the original image of 50 MHz SR. The recovered results of 50% CR, 60% CR and 70% CR are shown in Figure 15b–d, respectively. We can see that, for 50% CR, the recovery is quite good and almost no obvious error occurs. In the case of 60% CR, six defects can be easily identified, although there are some slight errors in the areas without defect echo. But when CR rise to 70%, the reconstruction is so distorted that some defects are almost overwhelmed by errors.

We also perform the PSNR comparison at different SRs when keeping the sampling points consistent. Table 4 reports the results using experimental data. For measurement points less than 220, the performance indeed becomes worse with the improvement of SR, which shows similar change rule compared with that in simulation. Besides, the gap of PSNRs narrows with measurement points’ rise and the difference disappears if measurement points are more than 240. It is therefore natural to confirm that lower M/N ratio affects the accuracy of OMP, while the reconstruction performance is independent with SR when measurement points increase to some extent.

**Figure 16:**
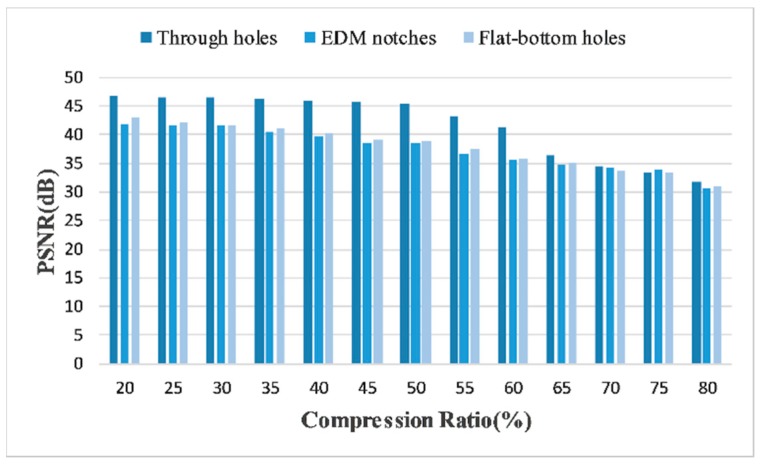
Comparative PSNRs between three kinds of defects in time domain, using 50 MHz SR experimental data.

To experimentally demonstrate the image quality in terms of various defects and evaluate its impact on reconstruction accuracy, we calculated and compared the PSNRs of three typical defects and showed the results in Figure 16. It turns out that, as expected, the recoveries of EDM notches and flat-bottom holes reach sufficiently good PSNRs, although suffer some reduction in lower CRs. Since the detected images of both two defects contain back wall echo or side echo, the preliminary analysis is that these strong echoes have a negative impact on the performances. 

**Figure 17:**
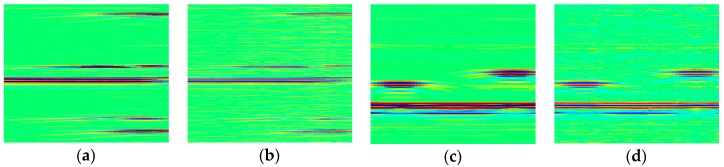
The original 50 MHz SR experimental image of (**a**) EDM notches, (**c**) flat-bottom holes and the reconstructed results in time domain, (**b**) CR = 60%, PSNR = 35.46 dB (**d**) CR = 60%, PSNR = 35.96 dB.

In Figure 17, we present the original images of EDM notches and flat-bottom holes, as well as the reconstructed results when CR is 60%. Although the image qualities, corresponding to the proposed CS method in time domain with OMP-based reconstruction, are reduced compared with the original versions, important information, for example, the measured defects, essential for structural health assessment, are all clearly preserved. 

### 4.3. Frequency Domain Reconstructions

**Figure 18:**
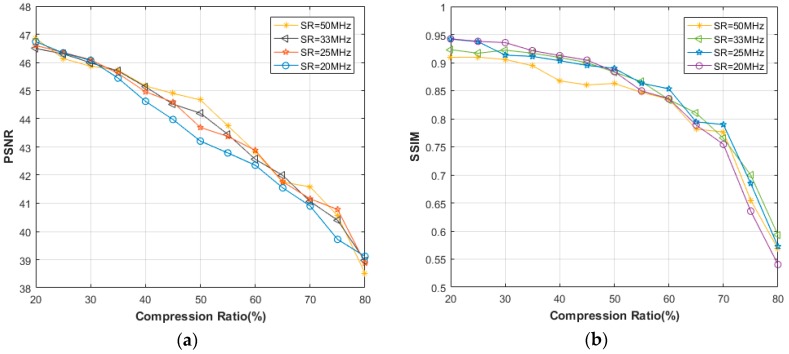
Comparative (**a**) PSNR, (**b**) SSIM between different SRs in frequency domain, using experimental through-holes data.

**Figure 19:**
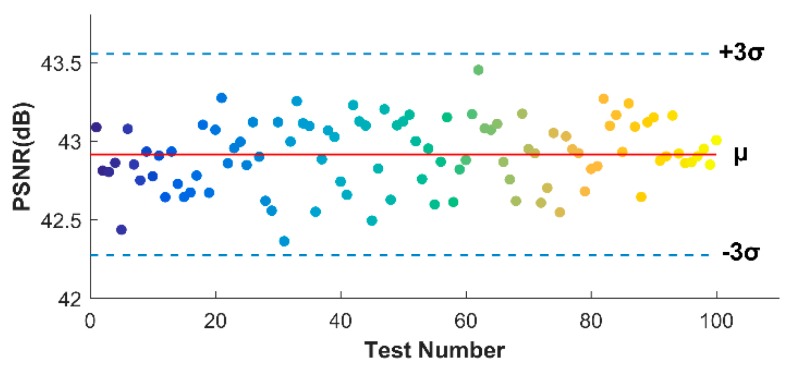
Distribution of 100 orthogonal matching pursuit (OMP) runs based on 3*σ* criterion of reliability theory in frequency domain, using 20 MHz SR experimental through-holes data when CR is 50%.

In this section, the performance of the frequency domain reconstruction is given and compared to the time domain results. Same as the simulation, the DCT transform is employed. Figure 18 shows the image calculations of different SRs. We first note that the performances, both PSNRs and SSIMs dramatically outperform the time domain results. To be more specific, the lowest PSNR in 60% CR is nearly 9.5 dB higher than that in time domain, and for SSIM, that is 0.49. When CR is 70%, the PSNR of 50 MHz SR image can be 41.61 dB, meaning a comparatively gratifying reconstruction. But for time domain, a 34.45 dB is obviously insufficient for defects recognition. Another observation is that the SR level in frequency domain seems no longer as significant as that in time domain. The PSNRs of 20 MHz SR are, maybe a little bit lower, not very far compared with 50 MHz SR, as for SSIMs, sometimes even better. Figure 19 shows the distributions of 100 OMP tests when 20 MHz SR experimental data is used and the CR is 50%, which completely reach the 3*σ* criterion of reliability theory. 

**Figure 20:**
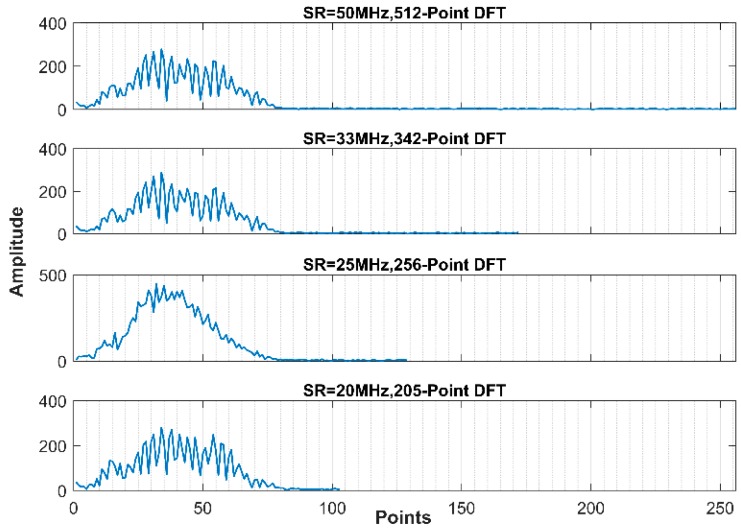
The frequency spectrums of different SRs.

We have explained in simulation section that the better performance in frequency domain, in comparison with time domain, owes to its higher sparsity. Now, according to the results in experimental case, we offer an interpretation on the performance similarity between different SRs. It is widely known that different SR brings about different sampling points. In time domain, the defect information locates in all the sampling points. When CR is given, lower SR means that the measurement points used for CS reconstruction are less, hence leads to poor results. Of course, we have confirmed through simulation and experiment that, if measurement points are not too few, recovery performance has nothing to do with SR. By contrast, in frequency domain, the ratio SR/N defines the frequency grid in DFT and N is always proportional to SR. So, the frequency grid is the same for all SRs here. Therefore, the points with relevant information of the useful band are the same for all SRs. To illustrate intuitively, the N-point DFT was applied to A-scan signals. We can see the point number of effective band retains almost the same level for different SRs, as illustrated in Figure 20. 

**Figure 21:**
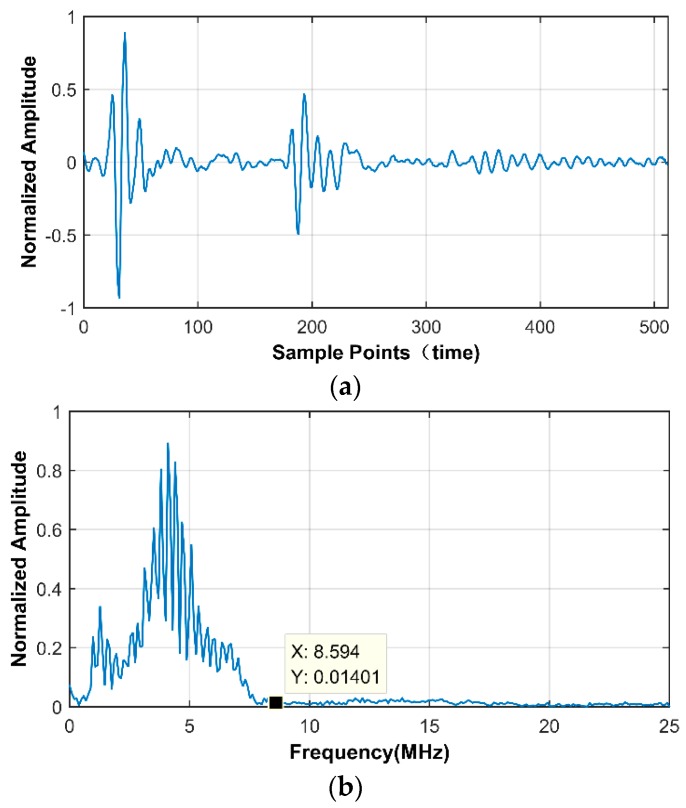
(**a**) Extracted A-scan signal from 20 MHz SR image; (**b**) the DFT spectrum of (**a**).

Before showing the frequency domain recovery performance of different kinds of defects, we show in Figure 21, one extracted A-scan signal from 20 MHz SR image and its corresponding DFT spectrum. It can be inferred that, using traditional sampling method, at least 17 MHz SR is required to avoid aliasing. Remarkably, it is possible to obtain satisfactory recovery using the equivalent of 10 MHz (for 50% CR), even 8 MHz (for 60% CR) samples, employing our proposed frequency domain CS scheme.

**Figure 22:**
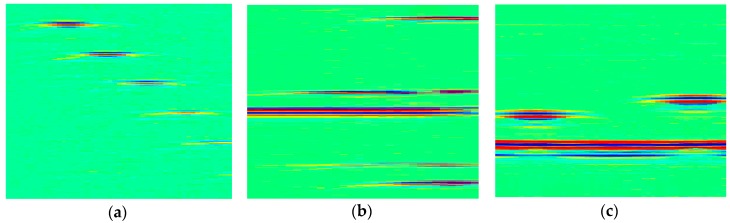
The recovery results of 20 MHz SR experimental image in frequency domain, (**a**) through-holes, PSNR = 42.91 dB; (**b**) EDM notches, PSNR = 41.50 dB; (**c**) flat-bottom holes, PSNR = 41.68 dB.

Figure 22 presents the recovery results of through-holes, EDM notches and flat-bottom holes, using 20 MHz SR image and 50% CR. The performances are totally comparable with that of 50 MHz SR in time domain, shown in previous section.

**Figure 23:**
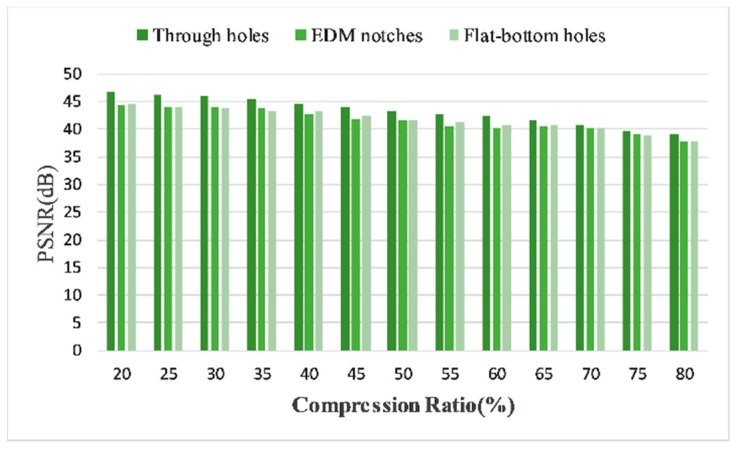
Comparative PSNRs between three kinds of defects in frequency domain, using 20 MHz SR experimental data.

Finally, in Figure 23 we show the comparative results of various defects. We can see that the performance disparities between different defects are smaller than that in time domain, which confirms the stabilization of the proposed DCT-based frequency domain reconstruction. As a conclusion, our proposed CS framework is able to drastically reduce the data in ultrasonic phased array imaging. What’s more, the results in frequency domain further strengthen the efficiency of CS, based on the fact that a breakthrough of the Nyquist sampling limitation is feasible.

### 4.4. Real Application in Engine Cylinder Cavity Inspection

As a case study, the proposed CS-based scheme was applied to the inspection of engine cylinder cavity in this part. 

**Figure 24:**
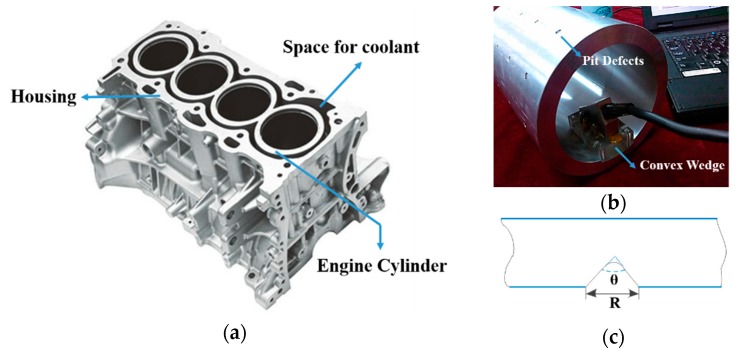
(**a**) The structure of engine cylinder; (**b**) The experimental system; (**c**) The details of pit defects.

The engine cylinder cavity plays the role of flowing the cooling liquid, as shown in Figure 24a. It can be corroded over time, which may bring about serious hidden dangers and have catastrophic consequences. To solve this problem, most of the researchers focused their studies on corrosion behavior or anticorrosion coolant. Despite the fact that these approaches can prevent the corrosions to an utmost degree, they are still inadequate in gaining the details of the flaws. Accordingly, routine detection can make some differences by identifying the potential risks and the ultrasonic phased array has been considered as an effective method for inspection of the cavity [32,33].

Considering the concave cylinder surface of the cavity, we designed a convex wedge installing on the phased array transducer. An aluminous specimen containing artificial pit defects, regarded as the most common defects caused by corrosion, was employed for our detection. The pits were machined with different diameters and taper angles in order to preserve the main characteristics of real defects. Figure 24b shows the experimental system and Figure 24c exhibits the details of a pit. The specific dimensions and angles of pit defects are tabulated in Table 5. It is noted that all the phased array configurations remain same with that in the last section.

**Figure 25:**
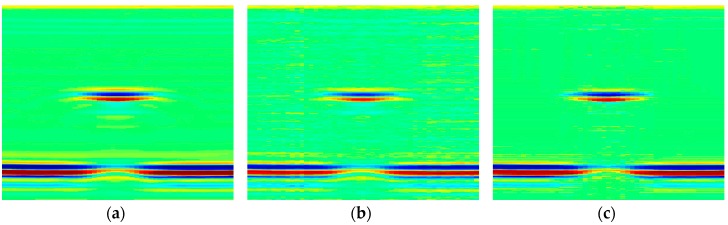
(**a**) The original 50 MHz SR experimental image of No. 3 pit; (**b**) the reconstructed image of (**a**) in time domain, CR = 60%, PSNR = 36.86 dB; (**c**) the recovery result in frequency domain, SR = 20 MHz, CR = 50%, PSNR = 40.84 dB.

In Figure 25, the original image of No. 3 pit and its reconstructed versions in time and frequency domain are given and compared. For time domain, 50 MHz SR image was used for the reconstruction and the CR is 60%. Figure 25b shows that, for this real application, the performance is generally satisfactory. Quite evidently, the recovery quality is much better in frequency domain, with the fact that lower measurement points (20 MHz SR, 50% CR) was used. A more important observation is that, from the reconstructed image shown in Figure 25c, frequency domain CS can inherently filter part background noises and enhance image quality.

**Figure 26:**
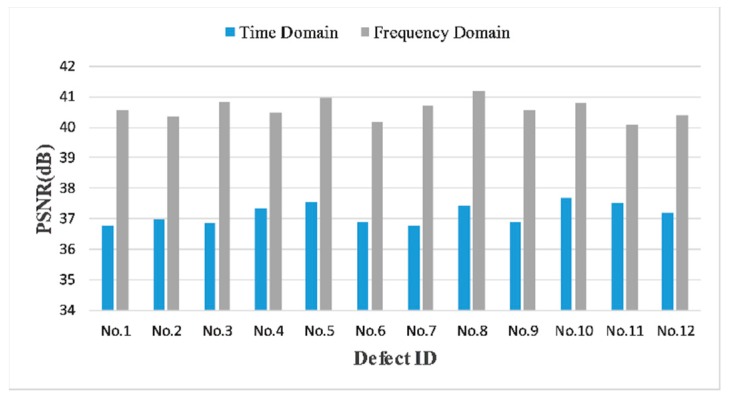
Comparative PSNRs between time and frequency domain for all pit defects, using 50 MHz SR, 60% CR in time domain and 20 MHz SR, 50% CR in frequency domain.

We show in Figure 26 the comparative PSNRs between time and frequency domain for all pit defects. Besides the obvious superiority of frequency domain reconstructions, the results indicate that the recovery performance is less likely to be affected by defects geometry, which validate the practicability of our proposed scenario in real application. 

## 5. Conclusions

Ultrasonic phased array imaging has always been a promising method for NDE applications because of its unique inspection flexibility and higher sensitivity. However, due to the increase of element numbers, a significant amount of data is acquired and needs to be processed. In this work, we proposed a CS-based data reduction framework and applied it into phased array image compression both in time field and frequency domain.

Based on CIVA platform, simulated images of different SRs were reconstructed and evaluated using both PSNR and SSIM index. In time domain, for 40 MHz SR (70% CR) or 25 MHz (60% CR), the recovery results are so gratifying that three defects are clearly preserved and can be easily identified, which suggest a breakthrough of the Nyquist sampling limitation. In frequency domain, the reconstruction performance is considerably better than that in time domain, with at least 49 dB PSNR for 70% CR. We offer an explanation from a perspective of “Information Value” and attribute the excellence to its higher sparsity of DCT coefficients. In addition, the results of different defects interval distance show that the proposed OMP-based CS scheme guarantees the reconstruction quality for close defects.

In experimental verification, images obtained from through-holes, EDM notches and flat-bottom holes are reconstructed when CR ranges from 20% to 80%. It is found that, in time domain, the recovery performances are relatively satisfactory when CR is 60% for 50 MHz SR, although not as good as that with simulated images. What’s more, the results are more liable to be affected by the SR level and we find it practically impossible to break Nyquist limitation. By contrast, both PSNRs and SSIMs in frequency domain appreciably outperform the time domain results with a 28.9% gain in PSNR and a 141% improvement in SSIM when SR is 20 MHz. For all three kinds of flaws, the coincident satisfactory recoveries confirm the stabilization of the proposed DCT-based frequency domain reconstruction. It is worth noting that, using the DCT coefficients to perform reconstruction, qualified recovery using the equivalent of 10 MHz samples can be obtained, which is below the sampling limitation and means a real breakthrough of the phased array data reduction. We also present that, in the real application of engine cylinder cavity inspection, the reconstruction performance keeps consistent for all pit defects with various diameters and taper angles, therefore verifies the enormous potential of our proposed scheme in the near future.

Besides, both in simulation and experiment, we compare the PSNR of different SRs when keeping the measurement points the same to find out whether SR truly influence the reconstruction performance. We find that lower M/N indeed has an adverse impact on the accuracy, because it leads to an ill-balanced sensing matrix. When the measurement points increase to some extent, however, the recoveries are independent with SR. The results imply that, rather than signal bandwidth, the success of CS relates the number of required measurements that carrying real information. That is to say, the sampling does not depend on signal bandwidth but on signal sparsity, which is the fundamental difference between CS and traditional sampling approach. Therefore, the conventional concept of “sampling rate” is no longer applicable in CS. It is merely because of the limitation of physical implementation, removing some parts of the original sample is used in this work.

## Figures and Tables

**Table 1:** Array parameters for simulation study.

Array Parameter	Value
Center Frequency	5 MHz
Element Count	64
Element Pitch	0.60 mm
Element Width	0.50 mm
Element Elevation	10.0 mm
Pulse Type	Gaussian weighted
−6 dB Bandwidth	50%

**Table 2:** Comparative PSNR of different SRs when keeping the same sampling points, using simulated data.

Measurement Points	200	220	240	260	280	300	320	340
20 MHz SR (584 points)	37.50	38.45	40.59	42.57	43.92	43.98	44.37	44.57
25 MHz SR (730 points)	34.99	37.56	37.65	41.06	43.83	44.19	44.23	44.44
30 MHz SR (876 points)	34.31	35.60	37.14	39.81	42.22	44.19	44.23	44.39
40 MHz SR (1168 points)	32.35	33.99	35.70	39.38	43.81	44.13	44.28	44.53

**Table 3:** The standard deviation values (*σ*) of different SRs and CRs in time domain (√ means the situation fits with 3*σ* criterion, X means not).

CR (%)		20	30	40	50	60	70	80
20 MHz SR	*σ*	0.4322	0.3878	0.4217	0.3930	0.4593	6.4072	9.3127
	Fit 3*σ* criterion	√	√	√	√	√	X	X
25 MHz SR	*σ*	0.3677	0.4539	0.3876	0.4211	0.4125	4.5018	5.3882
	Fit 3*σ* criterion	√	√	√	√	√	X	X
33 MHz SR	*σ*	0.4482	0.3975	0.4421	0.3903	0.3759	0.5225	3.5697
	Fit 3*σ* criterion	√	√	√	√	√	√	X
50 MHz SR	*σ*	0.3855	0.4203	0.3547	0.3971	0.3632	0.4125	3.4338
	Fit 3*σ* criterion	√	√	√	√	√	√	X

**Table 4:** Comparative PSNR of different SRs when keeping the same sampling points, using experiment data.

Measurement Points	160	180	200	220	240	260	280	300
25 MHz SR (256 points)	39.44	42.47	44.47	44.52	44.63	N/A	N/A	N/A
33 MHz SR (341 points)	36.61	41.04	42.10	43.32	43.89	44.68	45.19	45.31
50 MHz SR (512 points)	34.82	36.37	41.01	42.51	43.56	45.36	45.66	45.87

**Table 5:** The diameters (R) and taper angles (*θ*) of pit defects.

No.	1	2	3	4	5	6	7	8	9	10	11	12
R (mm)	2	3	4	5	2	3	4	5	2	3	4	5
*θ* (°)	120	120	120	120	90	90	90	90	60	60	60	60
